# Dieta Intermitente Atenua a Remodelação Cardíaca Causada pelo Exercício Físico

**DOI:** 10.36660/abc.20190349

**Published:** 2020-08-19

**Authors:** Priscilla Gois Basilio, Ana Priscila Cayres de Oliveira, Ana Carolini Ferreira de Castro, Marianna Rabelo de Carvalho, Alessandro Moura Zagatto, Paula Felippe Martinez, Marina Politi Okoshi, Katashi Okoshi, Gabriel Elias Ota, Filipe Abdalla dos Reis, Silvio Assis de Oliveira-Junior

**Affiliations:** 1 Laboratório de Estudo do Músculo Estriado Universidade Federal de Mato Grosso do Sul Campo Grande MS Brasil Laboratório de Estudo do Músculo Estriado (LEME/INISA), Universidade Federal de Mato Grosso do Sul,Campo Grande, MS - Brasil; 2 Laboratório de Fisiologia e Desempenho Esportivo Faculdade de Ciências Universidade Estadual Paulista Bauru SP Brasil Departamento de Educação Física, Laboratório de Fisiologia e Desempenho Esportivo (LAFIDE), Faculdade de Ciências - Universidade Estadual Paulista (UNESP),Bauru, SP - Brasil; 3 Faculdade de Medicina de Botucatu Departamento de Clínica Médica Universidade Estadual Paulista Botucatu SP Brasil Faculdade de Medicina de Botucatu - Departamento de Clínica Médica - Universidade Estadual Paulista (UNESP),Botucatu, SP - Brasil; 4 Centro Universitário Anhanguera de Campo Grande Campo Grande MS Brasil Centro Universitário Anhanguera de Campo Grande,Campo Grande, MS – Brasil

**Keywords:** Dieta Saudável, Restrição Calórica, Exercício Físico, Corrida, Remodelação Ventricular, Índice de Glicemia, Promoção da Saúde

## Abstract

**Fundamento:**

A influência de intervenções não farmacológicas como restrição calórica e exercício físico sobre a saúde e prevenção de enfermidades cardíacas tem sido documentada em estudos clínicos e experimentais.

**Objetivo:**

Analisar a influência da combinação entre dieta intermitente e exercício físico sobre a capacidade funcional, metabolismo glicêmico e remodelação cardíaca.

**Métodos:**

Foram utilizados 60 ratos Wistar machos distribuídos em quatro grupos: Controle (C), Exercício Físico (EF), Dieta Intermitente (DI) e Exercício Físico e Dieta Intermitente (EDI). Durante 12 semanas, enquanto C e EF foram tratados diariamente com dieta comercial padrão ad libitum, DI e EDI receberam dieta similar em dias alternados com dias de jejum. Os grupos EF e EDI foram submetidos a protocolo de corrida em esteira rolante. Posteriormente, foram analisadas capacidade funcional, comportamento nutricional e metabolismo glicêmico. Além da morfologia do coração, a expressão proteica das proteínas *extracellular signal-regulated kinase* (ERK) e *c-Jun N-terminal kinase* (JNK) no coração foi avaliada por Western-blot. A análise dos resultados foi feita por meio de *Two-Way* ANOVA e teste de Student-Newman-Keuls. O nível de significância considerado foi de 5%.

**Resultados:**

O exercício físico aumentou a capacidade funcional nos grupos EF e EDI, e acarretou fibrose cardíaca. A combinação entre dieta intermitente e exercício físico resultou em menor área sob a curva de glicemia e menores medidas de área e interstício cardíaco no EDI em relação ao EF. A expressão de proteínas ERK e JNK foi similar entre os grupos (p>0,05).

**Conclusões:**

Dieta intermitente se associa com melhor tolerância glicêmica e atenua o processo de remodelação cardíaca decorrente do exercício físico. (Arq Bras Cardiol. 2020; 115(2):184-193)

## Introdução

Classicamente, a restrição calórica é popularmente adotada como intervenção para melhorar a saúde, visando a promoção de benefícios funcionais ao organismo e maior longevidade.^1 - 4^ Entretanto, estudos experimentais têm mostrado respostas controversas no aspecto cardiovascular, pois a restrição calórica se mostrou associada com disfunção contrátil e danos morfológicos no miocárdio.^5 - 8^ Alguns pesquisadores constataram que a restrição calórica resultou em lesões de ultraestrutura miofibrilar e mudanças no trânsito intracelular de cálcio, relacionadas com distúrbios do sistema β-adrenérgico, contribuindo, assim, para disfunção contrátil do miocárdio.^5 , 7 , 9^ As modificações morfológicas envolveram também dilatação de câmaras ventriculares, degeneração dos cardiomiócitos, fibrose intersticial e edema mitocondrial.^10 - 12^

Além disso, após 12 semanas de experimento, o modelo intermitente de restrição calórica esteve pouco associado com danos morfológicos e não promoveu disfunção miocárdica, em comparação à restrição calórica de 50%.^11^ Na restrição calórica intermitente, o alimento é disponibilizado ad *libitum* em intervalos alternados com períodos de jejum, cada qual com duração de 12 a 24 horas.^3 , 4^ São escassos os estudos sobre os efeitos desse tipo de intervenção sobre o coração. No aspecto molecular, inclusive, a participação de proteínas cinases ativadas do mitógeno (MAPK), importantes agentes do processo de remodelação cardíaca,^13^ não foi ainda estudada em modelos de restrição calórica. As MAPKs incluem três subtipos principais, *extracellular signal-regulated* (ERK), *c-Jun N-terminal* (JNK) e p38 (p38K), que regulam a transcrição gênica de diversos mensageiros envolvidos na sobrevida, apoptose, diferenciação celular e remodelação cardíaca.^13 , 14^

Por sua vez, a prática regular de exercício físico é amplamente difundida como medida de promoção de saúde e prevenção de diferentes condições cardiovasculares^15 , 16^ Entretanto, diferentes estudos experimentais mostraram resultados controversos, evidenciando que o exercício físico não afetou e, inclusive, reduziu o desempenho miocárdico.^8 , 9 , 17 - 19^ Além disso, não foram encontradas evidências em relação à influência de protocolos de exercício físico em esteira rolante sobre o processo de remodelação cardíaca na restrição calórica intermitente.

Portanto, o objetivo do presente estudo foi analisar a influência da combinação entre restrição calórica intermitente e prática de exercício físico sobre o desempenho físico ao esforço e indicadores morfológicos e moleculares de remodelação miocárdica. Como hipótese inicial do presente estudo, admite-se que o exercício físico amplia o desempenho físico e atenua a remodelação miocárdica decorrente da restrição calórica intermitente.

## Métodos

O projeto científico foi analisado e aprovado pelo Comitê de Ética no Uso de Animais (CEUA/UFMS; Protocolo 615/2014), estando em conformidade com os regimentos do Colégio Brasileiro de Experimentação Animal (COBEA).

### Animais e Protocolo Experimental

Foram utilizados 60 ratos da linhagem *Wistar (Rattus novergicus albinus)* , machos, com 60 dias de idade, procedentes do Biotério Central da Universidade Federal de Mato Grosso do Sul (UFMS). A definição do tamanho amostral baseou-se em estudo prévio^16^ e considerou a probabilidade de recusa ao exercício e/ou instinto de fuga durante o teste de esforço.^20 , 21^ Utilizando-se de amostragem casual simples, os animais foram distribuídos em quatro grupos: Controle (C), Dieta Intermitente (DI), Exercício Físico (EF) e Exercício Físico e Dieta Intermitente (EDI). Enquanto CT e EF foram tratados diariamente com ração ad *libitum* (Nuvilab^®^, Brasil), os grupos DI e EDI receberam tratamento similar, administrado em dias alternados com dias de jejum.

Além do suporte nutricional, os animais dos grupos EF e EDI foram submetidos a um protocolo de corrida em esteira rolante ( Tabela 1 ) elaborado de acordo com estudos prévios.^16 , 20^ Foram realizadas cinco sessões semanais de exercício físico e o período experimental perdurou por 12 semanas. Os animais foram mantidos em gaiolas coletivas com duas a três unidades por caixa, sob temperatura ambiente de 22±2 ^º^C, umidade de 55±5%, ciclos de iluminação claro/escuro de 12 horas e água sob livre acesso.

**Tabela 1:** – Protocolo de exercício físico em esteira rolante, segundo período, velocidade média e duração das sessões

Período	Velocidade (m/min)	Duração (min)
1ª – 3ª semana	10	40– 60
4ª – 6ª semana	15	40
7ª – 9ª semana	18	35
10ª – 12ª semana	19	15– 25

### Teste de Esforço

Para analisar a capacidade funcional, ao término do experimento, realizou-se teste incremental de estágios múltiplos conforme estudos prévios.^21 , 22^ O teste foi iniciado com aquecimento de 5 minutos à velocidade de 5 m/min. Após 1 min de intervalo, cada animal foi submetido a um esforço progressivo, com velocidade inicial de 6 m/min, seguido por incrementos de 3 m/min, que perdurou por 3 minutos. O protocolo foi finalizado quando o animal atingisse a exaustão ou quando a coordenação entre as passadas se apresentasse dificultada.^21^

Para avaliar a resposta de lactato, 25 µl de sangue foram coletadas da cauda do animal em repouso e após cada estágio de esforço. O sangue coletado foi imediatamente armazenado em tubo Eppendorf contendo 50 µl de fluoreto de sódio (NaF) a 1%. As amostras de sangue foram refrigeradas após a coleta e, em seguida, mantidas em freezer (-20 °C) até a análise, que foi realizada em analisador eletroquímico YSI 150 Sport ( *Yellow Springs Instruments* ®, Ohio, EUA) com erro-padrão da medida de ±2%.

Os resultados foram dados em mmol/l e a determinação do limiar anaeróbico de lactato (LL) foi realizada por plotagem gráfica do comportamento das concentrações durante o teste. O LL foi determinado pelo momento de quebra da linearidade em função do aumento de carga, obtido mediante inspeção visual. A capacidade funcional foi avaliada pela velocidade no limiar de lactato (VLL), distância percorrida, concentração de lactato sanguíneo no limiar de lactato (LacLL) e no momento da exaustão (LacE), determinados durante o teste de esforço. Além disso, para melhor detalhar a cinética de lactato, considerou-se também a variação relativa (%) de níveis de lactato, obtida a partir das medidas de lactato LacLL e LacE.

### Caracterização Metabólica

Para análise do metabolismo glicêmico, os animais foram submetidos a jejum por 8–12 horas e amostras sanguíneas provenientes da artéria caudal foram utilizadas para a dosagem de glicose na condição basal. A seguir, realizou-se administração intraperitoneal de glicose a 20% (Glicose Monohidratada, Merck, São Paulo, Brasil), em dosagem equivalente a 2 g/kg. Os níveis glicêmicos foram então avaliados após 15, 30, 60, 90, 120 e 180 minutos.^7 , 10^ Para tanto, utilizou-se do glicosímetro ACCU-CHEK GO KIT (Roche Diagnostic Brazil Ltda, SP, Brasil).^23 , 24^

### Caracterização Nutricional

A caracterização nutricional envolveu ingestão alimentar (IA), ingestão calórica (IC) e eficiência energética. A IA foi avaliada diariamente e a IC foi calculada pela seguinte fórmula: IA × (valor calórico da dieta).^23^ A massa corporal foi mensurada semanalmente, utilizando-se uma balança digital. A variação ponderal foi obtida a partir da diferença entre os valores de massa corporal inicial e final, segundo momento de análise. Para analisar a capacidade de conversão da energia ingerida em massa corporal, considerou-se a eficiência alimentar (EA), obtida a partir da relação entre variação ponderal total (g) e energia total ingerida (kcal).^23 , 24^

Após o período experimental os animais foram mantidos em jejum por um período de oito horas, submetidos a anestesia intraperitoneal com cloridrato de cetamina (50 mg/kg/ip; Dopalen®, Sespo Indústria e Comércio Ltda – Divisão Vetbrands, Jacareí, São Paulo, Brasil) e cloridrato de xilazina (10 mg/kg/ip; Anasedan®, Sespo Indústria e Comércio Ltda – Divisão Vetbrands, Jacareí, São Paulo, Brasil). Após a eutanásia por decapitação, foram executadas toracotomia e laparotomia mediana para remoção do coração e retirada de tecido adiposo branco dos compartimentos retroperitoneal e epididimal.^24^ Considerou-se a soma dos dois compartimentos em valores absolutos e relativos para a determinação da adiposidade corporal.

### Caracterização Morfológica do Coração

Para avaliar a morfologia macroscópica do coração, foram mensuradas as massas de átrios (MA) e dos ventrículos direito (MVD) e esquerdo (MVE) em valores absolutos e em relação à massa corporal final (MCF) e o comprimento da tíbia. Posteriormente, foram retiradas amostras do ventrículo esquerdo a partir de incisão transversal a 6 mm do ápice. Os fragmentos foram imersos em solução tamponada de formol a 10%, na qual foram mantidos por 48 horas. Cada fragmento miocárdico foi então submetido a água corrente e mantidos sob solução de etanol a 70% por mais 48 horas. Após a etapa de fixação, os materiais foram compactados em blocos de parafina. Foram confeccionadas lâminas histológicas com secções teciduais de 4 a 7 μm de espessura, submetidas à coloração com hematoxilina-eosina (HE) e *picro-sirius* red (PSR). Para a análise morfométrica dos cardiomiócitos, foram consideradas medidas de área cardiomiocitária e fração intersticial de colágeno do miocárdio.^23 - 25^

Para a tomada de medidas de áreas, foram consideradas lâminas coradas em HE; para cada animal, foram amostrados pelo menos 100 cardiomiócitos. As lâminas coradas por PSR foram utilizadas somente para a quantificação do conteúdo de colágeno do meio intersticial miocárdico. Fixado o campo de imagem, os componentes do tecido cardíaco foram identificados segundo a cor realçada. Os filamentos de colágeno refletiram a cor vermelha enquanto os cardiomiócitos revelaram a coloração amarela. A fração intersticial de colágeno correspondeu à medida relativa (%) do conteúdo de colágeno sobre toda a extensão tecidual. Um mínimo de 20 campos foi utilizado e regiões perivasculares foram desconsideradas.

Como instrumental analítico, os cortes histológicos foram projetados em aumento de 40X com o auxílio de microscópio (LEICA DM LS) acoplado a uma câmera de vídeo que projeta imagens digitais em um microcomputador IBM equipado com programa analisador de imagens *Image Pro-plus* (Media Cybernetics, Silver Spring, Maryland, EUA).

### Análise da Expressão de MAPK

Os níveis de expressão proteica de MAPK foram determinados por meio de procedimentos de Western blot, utilizando-se anticorpos primários específicos (Santa Cruz Biotechnology Inc., CA, EUA): p-JNK (sc-6254), total JNK1/2 (sc-137019), p-ERK1/2 (sc-16982), total ERK 1 (sc-93). Os níveis de proteína obtidos foram normalizados pela expressão de GAPDH (6C5, sc-32233). Os métodos de preparação tecidual e condições de eletroforese são detalhados em estudos previamente publicados.^24 , 26^

### Análise Estatística

Para análise estatística dos resultados, utilizou-se o software Sigma Stat. Para estudar a distribuição de dados em relação à normalidade, utilizou-se o teste de Kolmogorov-Smirnov. Os resultados paramétricos foram apresentados em média e desvio-padrão e foram analisados com emprego de análise de variância de duas vias ( *Two-Way* ANOVA) complementada com teste de comparações de Student-Newman-Keuls. Os resultados de área celular foram distribuídos em categorias segundo intervalo de medidas, utilizando-se a fórmula de Sturges.^27^ Posteriormente, realizou-se análise de proporção absoluta e relativa com emprego de teste de proporções multinomiais de Goodman.^28^ Todas as conclusões estatísticas foram discutidas ao nível de significância de 5%.

## Resultados

**Figura 1:**
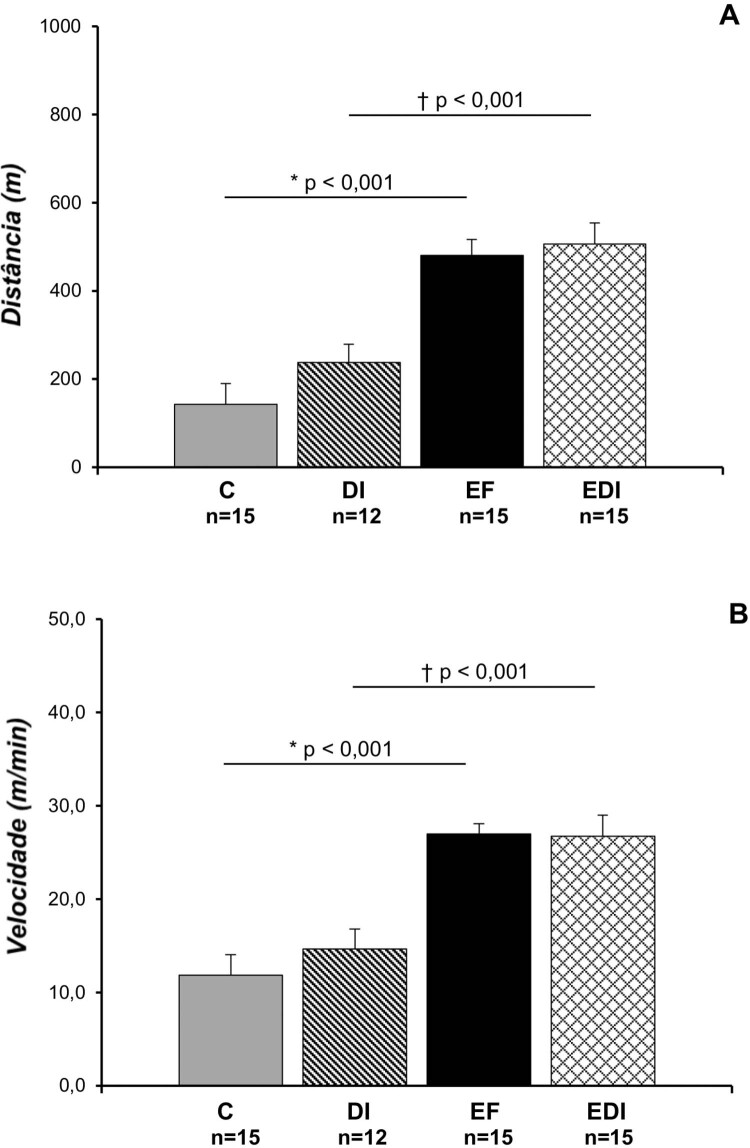
– Medidas de desempenho obtidas no teste de esforço, em média ± erro-padrão; (A) Distância Percorrida; (B) Velocidade; C: grupo Controle; DI: grupo Dieta Intermitente; EF: grupo Exercício Físico; EDI: grupo Exercício Físico e Dieta Intermitente. * p<0,001 vs. C; † p<0,001 vs. DI; Two-Way ANOVA e teste de Student-Newman-Keuls.

Na 1, são mostrados os valores da distância total (m) e velocidade final (m/min) obtidos no teste de esforço, que foi realizado no final do período experimental. Ambos os grupos exercitados, EF e EDI, apresentaram maiores valores de distância total e velocidade final, em comparação aos seus respectivos controles, C e DI. O efeito da dieta não foi significativo nas comparações efetuadas ( Figura 1A e 1B ).

Considerando-se as medidas finais de lactato, referente ao momento de exaustão (LacE), verificou-se efeito significativo (p=0,04) do exercício físico (C e DI: 8,16±0,94; EF e EDI: 5,34±0,88 mmol.L^-1^), sem ocorrência de interação fatorial. O limiar de lactato (LL) foi similar entre os grupos (C: 2,51±1,18; DI: 3,90±0,64; EF: 2,70±0,23; EDI: 3,04±1,33 mmol.L^-1^). A variação dos níveis de lactato entre o ponto de inflexão e o momento final do teste foi maior (p=0,04) nos grupos sedentários (C e DI: 156±19; EF e EDI: 98±18%).

**Figura 2:**
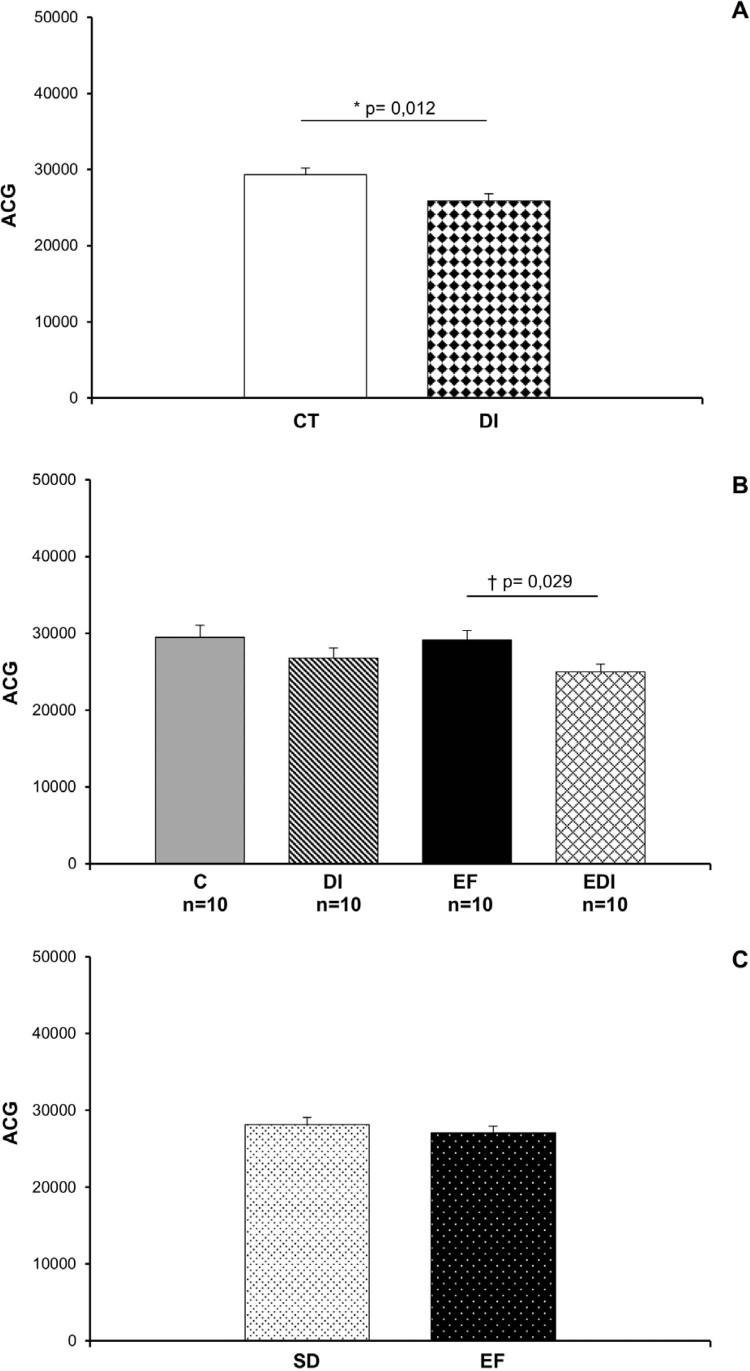
– Medidas de área sob a curva de tolerância glicêmica (ACG). (A) efeito isolado da dieta intermitente; CT: dieta controle ad libitum; DI: dieta intermitente; * p<0,05 versus CT. (B) efeito combinado: C, ratos sedentários sob dieta controle ad libitum; DI: ratos sedentários sob dieta intermitente; EF: ratos exercitados sob dieta controle ad libitum; EDI: ratos exercitados sob dieta intermitente, † p<0,05 versus EF; (C) efeito isolado do exercício físico; SD: grupos sedentários; EF: grupos exercitados. Two-Way ANOVA e teste de Student-Newman-Keuls

Na Figura 2 , são mostrados os valores referentes à área sob a curva de tolerância glicêmica. Considerando-se o efeito isolado da dieta ( Figura 2A ), constatou-se que a restrição intermitente se associou com menor área de resposta glicêmica. Não foram observadas diferenças significativas em relação ao efeito isolado do exercício ( Figura 2C ). No delineamento original ( Figura 2B ), o grupo EDI mostrou menor valor de área sob a curva glicêmica em relação ao EF. Não foram constatadas diferenças nas demais comparações entre grupos.

Em relação às variáveis nutricionais, as medidas de ingestão alimentar e consumo calórico foram menores nos grupos DI e EDI, quando comparados aos respectivos controles. Como fatores isolados, dieta intermitente e exercício físico resultaram em reduzida eficiência energética e menor ganho de massa corporal. Embora o grupo DI tenha mostrado menor massa corporal que o C, os valores de adiposidade não foram diferentes entre os grupos ( Tabela 2 ).

**Tabela 2:** – Características nutricionais e de morfologia cardíaca segundo grupo (dieta x exercício físico)

Características		Grupo				Fatores (p-valor)		
		C n=15	DI n=15	EF n=15	EDI n=15	Dieta	Condição	Interação
**Nutricionais**	**IA (g/dia)**	23,30 ± 0,88	17,64 ± 1,00 *	22,88 ± 0,65	17,88 ± 0,77 †	< 0,001	0,682	0,126
	**IC (kcal/dia)**	84,81 ± 3,20	64,20 ± 3,63 *	83,23 ± 2,38	65,10 ± 2,80 †	< 0,001	0,665	0,121
	**ICT (kcal/dia)**	7124 ± 269	5393 ± 305 *	6980 ± 192	5468 ± 236 †	< 0,001	0,602	0,100
	**EE (kcal/g)**	0,023 ± 0,005	0,019 ± 0,004 *	0,020 ± 0,004	0,017 ± 0,005	0,005	0,050	0,581
	**MC (g)**	395 ± 46	344 ± 37 *	374 ± 39	349 ± 30	< 0,001	0,400	0,202
	**VM (%)**	71,0 ± 17,8	42,8 ± 12,0 *	60,5 ± 15,2	37,4 ± 13,6 †	< 0,001	0,043	0,498
	**Adiposidade (%)**	2,11 ± 0,51	1,91 ± 0,77	1,89 ± 0,72	1,89 ± 0,79	0,584	0,508	0,578
**Morfologia Cardíaca**	**MA (g)**	0,059 ± 0,012	0,052 ± 0,009	0,066 ± 0,013	0,055 ± 0,015 †	0,006	0,126	0,422
	**MVD (g)**	0,134 ± 0,017	0,118 ± 0,034	0,145 ± 0,040	0,130 ± 0,020	0,055	0,143	0,950
	**MVE (g)**	0,482 ± 0,066	0,404 ± 0,041 *	0,469 ± 0,060	0,436 ± 0,059	< 0,001	0,509	0,138
	**MA/MC (mg/g)**	0,152 ± 0,030	0,152 ± 0,026	0,177 ± 0,036	0,155 ± 0,048	0,247	0,136	0,227
	**MVD/MC (mg/g)**	0,345 ± 0,033	0,346 ± 0,101	0,380 ± 0,076	0,368 ± 0,063	0,774	0,135	0,737
	**MVE/MC (mg/g)**	1,24 ± 0,14	1,18 ± 0,10	1,24 ± 0,09	1,23 ± 0,14	0,202	0,437	0,457
	**MA/CT (g/cm)**	0,015 ± 0,003	0,013 ± 0,002	0,017 ± 0,003	0,014 ± 0,004 †	0,007	0,227	0,328
	**MVD/CT (g/cm)**	0,034 ± 0,004	0,030 ± 0,009	0,036 ± 0,009	0,032 ± 0,004	0,065	0,246	0,982
	**MVE/CT (g/cm)**	0,121 ± 0,015	0,104 ± 0,009 *	0,117 ± 0,015	0,109 ± 0,015	< 0,001	0,892	0,170
	**Coração (g)**	0,674 ± 0,083	0,574 ± 0,063 *	0,680 ± 0,086	0,621 ± 0,058 †	< 0,001	0,172	0,292

IA: ingestão alimentar diária; IC: ingestão calórica diária; ICT: ingestão calórica total; EE: eficiência energética; MC: massa corporal; VM: variação relativa de massa corporal; MA: massa de átrios; MVD: massa de ventrículo direito; MVE: massa de ventrículo esquerdo; MA/MC: relação entre massa de átrios e massa corporal; MVD/MC: relação entre massa de ventrículo direito e massa corporal; MVE/MC: relação entre massa de ventrículo esquerdo e massa corporal; MA/CT: relação entre massa de átrios e comprimento da tíbia; MVD/CT: relação entre massa de ventrículo direito e comprimento da tíbia; MVE/CT: relação entre massa de ventrículo esquerdo e comprimento da tíbia. * p<0,05, comparado ao grupo C; † p<0,05 vs. grupo EF; Two-Way ANOVA e teste de Student-Newman-Keuls.

Considerando-se os resultados de morfologia cardíaca, a dieta intermitente, *per se* , resultou em menores valores de massa de átrios (0,063±0,002 vs. 0,053±0,002 g; p=0,006) e ventrículo esquerdo, em medidas absolutas (0,475±0,011 vs. 0,420±0,011 mg; p<0,001) e nas relações com o comprimento tibial. Além disso, a dieta reduziu a massa do coração (0,677±0,013 vs. 0,597±0,013 g; p<0,001) ( Tabela 2 ).

**Figura 3:**
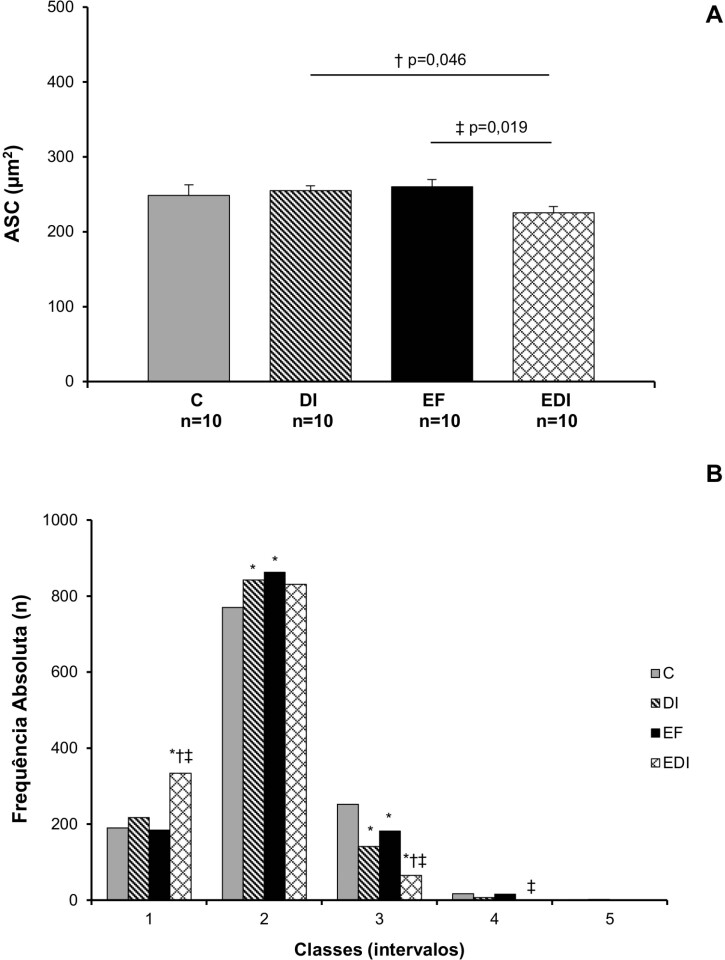
– (A) Área seccional transversa do cardiomiócito (ASC); † p<0,05 versus DI; ‡ p<0,05 versus EF. Two-Way ANOVA e teste de Student-Newman-Keuls. (B) Distribuição de frequências de cardiomiócitos segundo intervalo de classes de ASC; Classes: 1 (52,7┤190,1 µm^2^), 2 (190,1┤327,6 µm^2^), 3 (327,6┤465,0 µm^2^), 4 (465,0┤602,4 µm^2^) e 5 (602,4┤739,9 µm^2^); * p<0,05 versus C; † p<0,05 versus DI; ‡ p<0,05 versus EF. Teste de Goodman para contrastes dentro e entre populações multinomiais. C: Controle; DI: Dieta Intermitente; EF: Exercício Físico; EDI: Exercício Físico e Dieta Intermitente.

Na Figura 3 , são mostradas as medidas descritivas de morfometria do miocárdio. A combinação entre dieta intermitente e exercício físico resultou em menores valores de área celular no grupo EDI, que se mostrou diferente dos grupos EF e DI (C: 248±46; DI: 255±21; EF: 260±30; EDI: 225±26 μm^2^). Considerando-se a distribuição categórica de cardiomiócitos, a maior parte dos resultados situou-se nas duas primeiras classes, delimitadas até 327,5 µm^2^. No entanto, o grupo EDI revelou maior frequência de fibras na 1ª classe de valores (até 190,1 µm^2^), comparado os demais grupos (p<0,05; Figura 3B ).

**Figura 4:**
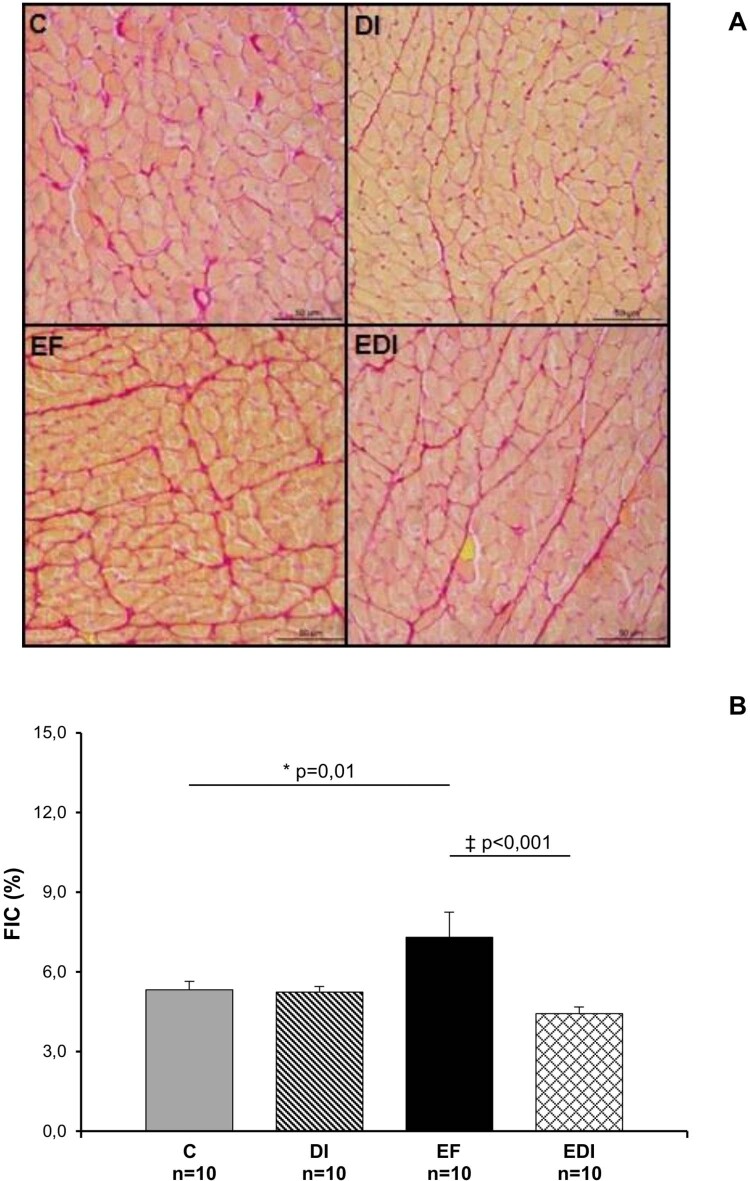
– (A) Seções transversas do miocárdio coradas com picro-sirius red, segundo o grupo; C: grupo Controle; DI: grupo Dieta Intermitente; EF: grupo Exercício Físico; EDI: grupo Exercício Físico e Dieta Intermitente. (B) Fração intersticial de colágeno (FIC); * p<0,05 versus C; ‡ p<0,05 versus EF. Two-Way ANOVA e teste de Student-Newman-Keuls

Em relação ao conteúdo de colágeno, observou-se interação estatisticamente significativa entre dieta e exercício físico (p=0,01). O grupo EF exibiu maior fração intersticial de colágeno em relação ao C (C: 5,32±1,02; DI: 5,25±0,66; EF: 7,31±2,94; EDI: 4,43±0,79%), enquanto o EDI apresentou menor concentração de colágeno que o EF ( Figura 4A-B ).

Na Tabela 3 , são apresentados os valores de expressão de proteínas MAPK ERK e JNK no miocárdio. Não foram constatadas diferenças em relação à expressão de proteínas MAPK, entre os grupos experimentais.

**Tabela 3:** – Expressão de proteínas MAPK do miocárdio, segundo grupo

Proteínas	Grupo			
	C n=6	DI n=6	EF n=6	EDI n=6
p-ERK/ERK	1,00 ± 0,52	1,42 ± 1,59	1,08 ± 0,48	1,23 ± 0,78
p-ERK/GAPDH	1,00 ± 0,47	1,18 ± 1,19	0,87 ± 0,41	0,86 ± 0,32
ERK/GAPDH	1,00 ± 0,10	0,99 ± 0,23	0,91 ± 0,16	0.91 ± 0,22
p-JNK/JNK	1,00 ± 0,39	1,00 ± 0,35	1,09 ± 0,61	1,03 ± 0,40
p-JNK/GAPDH	1,00 ± 0,13	1,11 ± 0,26	1,10 ± 0,35	1,15 ± 0,23
JNK/GAPDH	1,00 ± 0,46	1,04 ± 0,42	0,94 ± 0,31	0.96 ± 0,32

*Valores em média ± desvio padrão; ERK: extracellular signal-regulated kinase; JNK: c-Jun N-terminal kinase; Two-Way ANOVA (p>0,05).*

## Discussão

Dietas restritivas vêm sendo comumente utilizadas para reduzir o risco de doenças crônicas, como obesidade, diabetes tipo 2, dislipidemia e enfermidades cardiovasculares. No presente estudo, a dieta intermitente se associou com maior consumo alimentar e energético nos dias de oferta, menor ingestão calórica total e menores medidas de massa corporal. Maior consumo alimentar decorrente da dieta intermitente pode ser explicado por alterações na sensação de saciedade.^29 , 30^ O hipotálamo é um dos principais responsáveis pela homeostase corporal, exercendo diversas funções, dentre elas, a promoção de saciedade. Sabe-se que alterações no hipotálamo lateral levam à afagia (inanição), enquanto desordens no hipotálamo medial conduzem à hiperfagia (aumento do apetite). Outros estudos^11 , 29^ mostraram que a dieta intermitente acarretou maior consumo alimentar nos dias de oferta, como apresentado no presente trabalho. Da mesma forma, Dorighello et al.,^31^ mostraram que a restrição intermitente resultou em menor ingestão calórica total, corroborando nossos achados. A vigência do exercício físico não afetou as respostas do consumo alimentar e calórico decorrentes da dieta. Buthani et al.,^32^ em estudo com humanos, demonstraram que voluntários exercitados, mesmo apresentando aumento de fome, não exibiram significativo aumento da ingestão alimentar.

Segundo a hipótese inicial do presente estudo, o exercício físico em esteira rolante atenua prováveis desordens metabólicas e o processo de remodelação cardíaca decorrentes da restrição calórica intermitente. Além de reduzir a eficiência energética, a dieta intermitente modificou a tolerância glicêmica, o que pode estar associado com melhor sensibilidade à ação insulinêmica. A insulina tem propriedades lipogênicas sobre o tecido adiposo,^33^ fato que poderia explicar porque os grupos sob dieta intermitente, mesmo com massa corporal inferior, não apresentaram diferenças significativas de adiposidade corporal, quando comparados aos respectivos controles ( Tabela 2 ). A insulina aumenta sua liberação quando há maior oferta de nutrientes, como no período pós-prandial. A melhora da sensibilidade à insulina pela adoção da dieta intermitente foi encontrada também em estudo recente com ratos.^34^

Por sua vez, a combinação entre dieta intermitente e exercício físico resultou em menor adiposidade corporal no EDI. O exercício físico promove adaptações e ajustes de natureza cardiorrespiratória, neural e hormonal.^9 , 15 , 16^ No contexto hormonal, a secreção dos hormônios é também alterada pelo exercício. No estudo de Evans et al.,^35^ a prática de exercício físico aeróbio durante 12 meses resultou em melhora na sensibilidade à insulina, com diminuição de 19,4% da área da curva de tolerância oral a glicose, em idosos. Portanto, a interação entre dieta e exercício pode ter potencializado os efeitos hormonais do metabolismo insulinêmico, como sustentado pelos achados nos grupos EF e EDI.

Apesar das repercussões no metabolismo glicêmico, a dieta intermitente não interferiu na capacidade funcional. Para a determinação do limiar anaeróbio, a mensuração dos níveis de lactato é um dos parâmetros mais utilizados para estimar a capacidade aeróbia e tem se mostrado um eficaz índice de avaliação desta capacidade.^22^ O limiar de lactato pode ser definido como a intensidade de exercício em que a concentração sanguínea de lactato tem um aumento abrupto.^21 , 22^ Nesse sentido, o protocolo de corrida resultou em melhor desempenho funcional dos grupos EF e EDI, o que foi sustentado por menores valores de lactato final (LacE), menor variação dos níveis de lactato e maiores valores de velocidade e distância percorrida durante o teste final. Portanto, pode-se afirmar que o exercício físico promoveu melhora da capacidade funcional, como já demonstrado.^16^

No aspecto cardiovascular, o exercício físico acarretou remodelação intersticial do miocárdio. Intrigantemente, a restrição intermitente promoveu retenção desses efeitos do exercício, o que foi sustentado por menores valores de morfometria macro e microscópica tecidual no EDI. Entre os fatores estimulantes da proliferação e estimulação do processo de remodelação miocárdica, incluem-se distúrbios nutricionais, angiotensina, aldosterona, endotelinas, citocinas inflamatórias e catecolaminas.^36^ Com a sobrecarga física prolongada, ocorrem alterações morfofuncionais no miocárdio, com o intuito de melhorar o desempenho do coração no bombeamento de sangue e na capacidade do sistema cardiovascular de fornecer oxigênio aos músculos recrutados durante o esforço.^21 , 22^ Entre essas adaptações do exercício, inclui-se a hipertrofia ventricular esquerda, desenvolvida para compensar a demanda hemodinâmica, e a fibrose intersticial.^36 , 37^ Nessa perspectiva, o aumento de colágeno intersticial encontrado no grupo EF, pode se configurar como indício do processo de remodelação ventricular fisiológica, ainda que os achados morfológicos não tenham confirmado a hipertrofia. Alguns fatores podem restringir a precisão da morfometria microscópica, incluindo-se variabilidade do ângulo de corte tecidual, estado contrátil heterogêneo das fibras cardíacas, entre outros.^38^ Tais condições podem ter contribuído para a não detecção de hipertrofia cardíaca derivada do exercício físico.

Por sua vez, restrição intermitente acarretou menores valores de morfologia macro e microscópica, sem afetar a expressão e ativação de proteínas ERK e JNK. As alterações fenotípicas carreadas por esses peptídeos envolvem síntese de proteínas e crescimento celular, com instalação de hipertrofia e fibrose intersticial, as quais podem ser associadas ao processo de remodelação miocárdica.^13 , 14^ Além disso, a ativação de MAPK é também subordinada à ação de fatores de crescimento, como hormônio do crescimento e insulina,^24 , 33^ os quais têm sua secreção regulada pelo comportamento nutricional. Apesar disso, não foi possível verificar a associação entre dieta intermitente e alteração na expressão de MAPK. Em estudo prévio.^39^ a dieta intermitente reduziu a hipertrofia cardíaca e a dilatação ventricular em ratos infartados, embora não tenha alterado a expressão gênica de peptídeos fetais.

Com o presente estudo, tem-se, portanto, a comprovação de que a remodelação cardíaca derivada do exercício físico foi amenizada pela dieta intermitente. Entretanto, não é possível afirmar se esse potencial profilático da intervenção dietética pode ser utilizado na reversão de processos patológicos, como na hipertensão arterial e infarto agudo do miocárdio. Da mesma forma, o impacto de outros modelos experimentais, incluindo-se 25 e 50% de restrição calórica^5 , 7 - 9^ deve ser melhor estudado em futuras investigações. Tais fatos trazem limitações em termos de repercussões clínicas para os resultados da presente investigação.

## Conclusão

A combinação de dieta intermitente e exercício físico se associa com maior tolerância glicêmica. O exercício físico, isoladamente, causa remodelação intersticial do miocárdio, que é atenuada pela intervenção com dieta intermitente.
