# Supplementary material for: Dieta Intermitente Atenua a Remodelação Cardíaca Causada pelo Exercício Físico
Source: Arq Bras Cardiol. 2020 Aug 19;115(2):184–93. [Article in Portuguese] doi: 10.36660/abc.20190349 (PMC8384291; doi:10.36660/abc.20190349)
Supplement: Translation

# Translation: Intermittent Fasting Attenuates Exercise Training-Induced Cardiac Remodeling

**Authors:** Priscilla Gois Basilio, Ana Priscila Cayres de Oliveira, Ana Carolini Ferreira de Castro, Marianna Rabelo de Carvalho, Alessandro Moura Zagatto, Paula Felippe Martinez, Marina Politi Okoshi, Katashi Okoshi, Gabriel Elias Ota, Filipe Abdalla dos Reis, Silvio Assis de Oliveira-Junior

**Affiliations:** 1 Brazil Laboratory of Striated Muscle Study (LEME/ INISA), Federal University of Mato Grosso do Sul, Campo Grande, MS - Brazil; 2 Brazil Department of Physical Education, Laboratory of Physiology and Sport Performance (LAFIDE), School of Sciences, São Paulo State University, UNESP, Bauru, SP - Brazil; 3 Brazil Botucatu Medical School, São Paulo State University, UNESP, Botucatu, SP - Brazil; 4 Brazil Centro Universitário Anhanguera de Campo Grande, Campo Grande, MS – Brazil

**Keywords:** Diet, Calorie Restriction, Exercise, Running, Ventricular Remodeling, Glucose, Health Promotion

## Abstract

**Background:**

The effects of non-pharmacological interventions such as calorie restriction and exercise training on health and prevention of cardiovascular diseases have been investigated in clinical and experimental studies.

**Objective:**

To analyze the influence of intermittent fasting and exercise training on functional fitness, glycemia and cardiac remodeling.

**Methods:**

Wistar rats (n=60) were randomly divided into four groups: control, exercise training (ET), intermittent fasting (IF) and exercise training plus intermittent fasting (ETI). Over 12 weeks, control and ET animals were fed daily a standard commercial diet ad *libitum* , while IF and ETI animals were fed every other day. In addition, the ET and ETI groups were submitted to a running protocol on a treadmill. After this period, functional fitness, nutritional parameters and blood glucose levels were analyzed. In addition to heart morphology, myocardial protein expression of extracellular signal-regulated kinase (ERK) and c-Jun N-terminal kinase (JNK) was assessed by Western-blot. The results were analyzed using two-way ANOVA and Student-Newman-Keuls test. The level of significance considered was 5%.

**Results:**

Exercise training increased functional fitness in the ET and ETI groups and promoted cardiac fibrosis. The combination of intermittent fasting and exercise training resulted in a smaller area under the blood glucose curve and reduced cardiomyocyte cross-sectional area and interstitial collagen fraction in the ETI group compared to ET. ERK and JNK expression levels were similar among groups (p>0.05).

**Conclusions:**

Intermittent fasting is associated with improved glucose tolerance and attenuates cardiac remodeling induced by exercise training (Arq Bras Cardiol. 2020; 115(2):184-193)

## Introduction

Classically, calorie restriction is a popular intervention for health improvement, promoting multiple functional benefits and increasing human longevity.^1 - 4^ However, experimental studies have reported controversial changes in cardiovascular parameters in response to calorie restriction, which was found to be associated with contractile dysfunction and myocardial morphological damage.^5 - 8^ Some researchers demonstrated that calorie restriction promoted ultrastructural injuries to cardiac myofibrils and changes in intracellular calcium handling; these responses were related to β-adrenergic system disorders and myocardial contractile dysfunction.^5 , 7 , 9^ Other morphological changes included ventricular chamber dilation, cardiomyocyte necrosis, interstitial fibrosis and mitochondrial swelling.^10 - 12^

On the other hand, intermittent fasting (IF) intervention, a model of calorie restriction, was associated with few morphological changes and did not promote myocardial dysfunction after a period of 12 weeks compared to 50% calorie restriction.^11^ In this dietary approach, food is available ad *libitum* at intervals alternating with fasting periods, each lasting 12 to 24 hours.^3 , 4^ However, the effects of IF on the heart remain unknown. At the molecular level, the association of mitogen-activated protein kinases (MAPKs), important mediators of cardiac remodeling,^13^ has not been studied in calorie restriction models. MAPKs include three main subtypes, extracellular signal-regulated kinase (ERK), c-Jun N-terminal kinase (JNK) and p38 kinase (p38K), which regulate the gene transcription of several messengers involved in survival, apoptosis, cell differentiation and cardiac remodeling.^13 , 14^

Regular exercise training is widely used as a procedure of health promotion and prevention of different cardiovascular conditions.^15 , 16^ However, different experimental studies have reported controversial results, showing that exercise training intervention did not affect or even reduced myocardial performance.^8 , 9 , 17 - 19^ Moreover, potential effects of treadmill training on cardiac remodeling in IF models have yet to be clarified. Therefore, the aim of this study was to analyze the influence of IF and exercise training on functional fitness and morphological and molecular parameters of myocardial remodeling. Our initial hypothesis is that exercise training increases functional fitness and attenuates myocardial remodeling induced by IF.

## Methods

The experimental protocol was reviewed and approved by the Animal Ethics Committee of the Federal University of Mato Grosso do Sul (CEUA/UFMS; Protocol 615/2014), and was in accordance with the guidelines of the Brazilian Society of Animal Experimentation (COBEA).

### Animals and Experimental Design

Male Wistar rats ( *Rattus norvegicus albinus* ; n=60), aged 30, obtained from the Animal Center of the Federal University of Mato Grosso do Sul (UFMS), Campo Grande/MS, Brazil, were used. The sample size was based on a previous study^16^ and considered the probability of exercise refusal and/or escape instinct during the test.^20 , 21^ The animals were randomly divided into four groups: control, intermittent fasting (IF), exercise training (ET) and exercise training plus intermittent fasting (ETI). The control and ET groups were fed daily ( *ad libitum* ) a standard commercial diet (Nuvilab®, Brazil), while IF and ETI received a similar dietary intervention every other day (intermittent fasting).

Besides nutritional support, animals of the ET and ETI groups were also submitted to a treadmill running protocol ( Table 1 ), according to previous studies.^16 , 20^ Five weekly exercise sessions of physical exercise were held and the experimental period lasted 12 weeks. The animals were kept in cages with two to three experimental units per box at an ambient temperature of 22±2 ºC, humidity of 55±5%, 12-hour light/dark cycle and free access to water.

**Table 1:** – Description of the treadmill exercise protocol

Period	Speed (m/min)	Duration (min)
Week 1–3	10	40–60
Week 4–6	15	40
Week 7–9	18	35
Week 10–12	19	15–25

### Determination of Functional Fitness

Functional fitness was analyzed at the end of the experiment using a multistage incremental test, according to previous studies.^21 , 22^ The test was started with a 5-minute warm-up at a speed of 5 m/min. After an interval of 1 min, each animal was submitted to progressive effort at an initial speed of 6 m/min, followed by increments of 3 m/min, lasting 3 min. The protocol was interrupted when the animal had reached exhaustion or when coordination between steps was difficult.^21^

For the evaluation of lactate levels, 25 µl of blood was collected from the animal’s tail at rest and after each exercise stage. The blood samples were immediately transferred to Eppendorf tubes containing 50 µl of 1% sodium fluoride (NaF), refrigerated, and stored in a freezer (-20 °C) until the time of analysis. Lactate was measured in an YSI 150 Sport electrochemical analyzer (Yellow Springs Instruments®, Ohio, USA), with standard error of the measurement of ± 2%.

The results are reported in mmol/L. The anaerobic lactate threshold (LT) was determined by graphically plotting lactate concentrations during the test. The LT was determined from the time of linearity breaking as a function of load increase, obtained by visual inspection. Functional capacity was assessed by the speed at the lactate threshold (SLT), distance covered and blood lactate concentration at the lactate threshold (LacLT) and at exhaustion (LacE), determined during the exercise test. In addition, for a more detailed analysis of lactate kinetics, the relative variation (%) in lactate levels was also obtained from the LacLT and LacE values.

### Metabolic Characterization

For analysis of glycemia, the animals were fasted for 8–12 hours and blood samples were collected from the caudal artery to measure glucose at baseline. Next, a 20% glucose solution (Glucose Monohydrate, Merck, São Paulo, Brazil) was administered intraperitoneally at a dosage of 2 g/kg. Blood glucose levels were then evaluated after 15, 30, 60, 90, 120 and 180 minutes.^7 , 10^ Glucose was measured with an Accu-Chek Go glucose meter (Roche Diagnostic Brazil Ltda., São Paulo, Brazil).^23 , 24^

### Nutritional Characterization

Nutritional characterization consisted of the measurement of food intake, calorie intake, and feed efficiency. Food intake was measured daily and calorie intake was calculated from food intake × energy density of the diet.^23^ Body weight (BW) was measured once a week using a digital scale. BW gain was obtained as the difference between initial and final BW. To analyze the ability to convert ingested energy into BW, feed efficiency was calculated as the ratio between total BW variation (g) and total calorie intake (kcal).^23 , 24^

After the experimental period, the animals were fasted for 8 hours and anesthetized by intraperitoneal administration of ketamine hydrochloride (50 mg/kg; Dopalen®, Sespo Indústria e Comércio Ltda — Vetbrands Division, Jacareí/SP, Brazil) and xylazine hydrochloride (10 mg/kg; Anasedan®, Sespo Indústria e Comércio Ltda — Vetbrands Division, Jacareí/SP, Brazil). After euthanasia by decapitation, thoracotomy and median laparotomy were performed in order to remove heart and white adipose tissue from the retroperitoneal and epididymal compartments.^24^ The sum of the two adipose sites in absolute and relative values was considered for the determination of body adiposity.

### Characterization of Cardiac Morphology

To assess macroscopic morphology, the atrial and left and right ventricular weights were measured in absolute values and compared to BW and tibia length. Samples of the left ventricle were obtained through a transverse incision 6 mm from the apex. The myocardial fragments were immersed in 10% buffered formaldehyde for 48 hours. Each tissue specimen was then rinsed under running water and stored in 70% ethanol solution for more 48 hours. After the fixation step, the specimens were embedded in paraffin blocks. Histological slides were prepared from 4–7 µm thick tissue cross-sections and stained with hematoxylin/eosin and picrosirius red. For the morphometric analysis of cardiomyocytes, the cardiomyocyte area and the myocardial interstitial collagen fraction were measured as described previously.^23 - 25^

Hematoxylin/eosin-stained slides were used for the measurement of cardiomyocyte area. At least 100 cardiomyocytes were sampled per animal. Picrosirius red-stained slides were used to determine interstitial collagen fraction. Once the image field was fixed, components of the cardiac tissue were identified according to the highlighted color. Collagen filaments appeared red, while cardiomyocytes appeared yellow. The interstitial collagen fraction corresponds to the percentage of collagen content throughout the tissue specimen. A minimum of 20 fields were used and perivascular regions were disregarded.

For myocardial morphometry, the histological sections were analyzed at a 40-fold magnification with a microscope (LEICA DM LS) coupled to a digital video camera on an IBM microcomputer, equipped with the image analyzer program Image Pro-plus (Media Cybernetics, Silver Spring, Maryland, USA).

### Analysis of MAPK Expression

MAPK protein expression levels were determined using Western blot procedures and specific primary antibodies (Santa Cruz Biotechnology Inc., CA, USA): p-JNK (sc-6254), total JNK1/2 (sc -137019), p-ERK1/2 (sc-16982), total ERK 1 (sc-93). The protein levels obtained were normalized to the expression of GAPDH (6C5, sc-32233). The sample preparation methods and electrophoresis conditions have been described previously.^24 , 26^

### Statistical Analysis

The Sigma-Stat software was used for data analysis. Firstly, the results were subjected to normality analysis by the Kolmogorov-Smirnov test. Since the variables had a parametric distribution, measures are presented as mean and standard-deviation and were analyzed using two-way analysis of variance (two-way ANOVA), complemented by the Student-Newman-Keuls comparison test. The cross-sectional area results of cardiomyocytes were divided into categories according to the measurement range using Sturges formula.^27^ Absolute and relative proportions were analyzed using the Goodman multiple proportions test.^28^ The level of significance was set at 5%.

## Results

The results of the functional fitness exercise on a treadmill [total distance (m) and final speed (m/min)] are shown in Figure 1 . Exercise groups ET and ETI achieved greater total distance and final speed than control and IF animals. Intermittent fasting did not affect functional fitness (p>0.05) ( Figures 1A and 1B ).

**Figure 1:** – Functional fitness measurements expressed as mean ± standard error; (A) distance covered; (B) final speed; Control: sedentary rats receiving the control diet ad libitum; IF: sedentary rats undergoing intermittent fasting; ET: rats exercising and receiving the control diet ad libitum; ETI: rats exercising while intermittent fasting. * p<0.05 vs. control; † p<0.05 vs. IF (two-way ANOVA and Student-Newman-Keuls test).

Regarding results of lactate measurements, exercise training exerted a statistically significant effect (p=0.04) on LacE (control and IF: 8.16±0.94; ET and ETI: 5.34±0.88 mmol×L^-1^), without the occurrence of factor interaction. The LT was similar among groups (control: 2.51±1.18; IF: 3.90 ± 0.64; ET: 2.70±0.23; ETI: 3.04±1.33 mmol×L^-1^). The variation in lactate levels between the inflection point and the end of the test was greater (p=0.04) in the sedentary groups (control and IF: 156±19; ET and ETI: 98±18%).

The areas under the glucose tolerance curve are shown in Figure 2 . Considering the individual effect of dietary intervention ( Figure 2A ), intermittent fasting was associated with smaller areas of glycemic response. There were no significant differences in the individual effect of exercise training ( Figure 2C ). On the other hand, ETI animals showed a smaller area under the glucose curve than ET ( Figure 2B ). No differences were found for the other statistical comparisons.

**Figure 2:** – Area under the glucose tolerance curve (AUC), according to condition and dietary intervention. (A) Individual effect of intermittent fasting; CT: groups submitted to control diet; IF: groups submitted intermittent fasting; *p<0.05 vs. CT. (B) Combined effect. Control: sedentary rats receiving the control diet ad libitum; IF: sedentary rats undergoing intermittent fasting; ET: rats exercising and receiving the control diet ad libitum; ETI: rats exercising while intermittent fasting; † p<0.05 vs. ET. (C) Individual effect of physical exercise training. SD: sedentary groups; ET: exercised groups Two-way ANOVA and Student-Newman-Keuls test.

With respect to nutritional variables, food consumption and caloric intake were lower in the IF and ETI groups compared to the respective controls. As individual factors, intermittent fasting and exercise training were associated with reduced energy efficiency and lower BW gain. Although BW gain was lower in the IF group compared to control, adiposity levels were similar between the groups ( Table 2 ).

**Table 2:** – Nutritional characteristics and cardiac morphology

Characteristics		Group				Factor (p-value)		
		Control n=15	IF n=15	ET n=15	ETI n=15	Diet	Exercise	Interaction
**Nutritional variables**	**FC (g/day)**	23.30 ± 0.88	17.64 ± 1.00 *	22.88 ± 0.65	17.88 ± 0.77 ‡	<0.001	0.682	0.126
	**CI (kcal/day)**	84.81 ± 3.20	64.20 ± 3.63 *	83.23 ± 2.38	65.10 ± 2.80 ‡	<0.001	0.665	0.121
	**TCI (kcal/day)**	7124 ± 269	5393 ± 305 *	6980 ± 192	5468 ± 236 ‡	<0.001	0.602	0.100
	**FE (kcal/g)**	0.023 ± 0.005	0.019 ± 0.004 *	0.020 ± 0.004	0.017 ± 0.005	0.005	0.050	0.581
	**BW (g)**	395 ± 46	344 ± 37 *	374 ± 39	349 ± 30	<0.001	0.400	0.202
	**BWV (%)**	71.0 ± 17.8	42.8 ± 12.0 *	60.5 ± 15.2	37.4 ± 13.6 ‡	<0.001	0.043	0.498
	**Adiposity (%)**	2.11 ± 0.51	1.91 ± 0.77	1.89 ± 0.72	1.89 ± 0.79	0.584	0.508	0.578
**Morphological variables**	**AW (g)**	0.059 ± 0.012	0.052 ± 0.009	0.066 ± 0.013	0.055 ± 0.015 ‡	0.006	0.126	0.422
	**RVW (g)**	0.134 ± 0.017	0.118 ± 0.034	0.145 ± 0.040	0.130 ± 0.020	0.055	0.143	0.950
	**LVW (g)**	0.482 ± 0.066	0.404 ± 0.041 *	0.469 ± 0.060	0.436 ± 0.059	<0.001	0.509	0.138
	**AW/BW (mg/g)**	0.152 ± 0.030	0.152 ± 0.026	0.177 ± 0.036	0.155 ± 0.048	0.247	0.136	0.227
	**RVW/BW (mg/g)**	0.345 ± 0.033	0.346 ± 0.101	0.380 ± 0.076	0.368 ± 0.063	0.774	0.135	0.737
	**LVW/BW (mg/g)**	1.24 ± 0.14	1.18 ± 0.10	1.24 ± 0.09	1.23 ± 0.14	0.202	0.437	0.457
	**AW/tibia (g/cm)**	0.015 ± 0.003	0.013 ± 0.002	0.017 ± 0.003	0.014 ± 0.004 ‡	0.007	0.227	0.328
	**RVW/tibia (g/cm)**	0.034 ± 0.004	0.030 ± 0.009	0.036 ± 0.009	0.032 ± 0.004	0.065	0.246	0.982
	**LVW/tibia (g/cm)**	0.121 ± 0.015	0.104 ± 0.009 *	0.117 ± 0.015	0.109 ± 0.015	<0.001	0.892	0.170
	**Heart weight (g)**	0.674 ± 0.083	0.574 ± 0.063 *	0.680 ± 0.086	0.621 ± 0.058 ‡	<0.001	0.172	0.292

FC: daily food consumption; CI; daily calorie intake; TCI: total calorie intake; FE: feed efficiency; BW: body weight; BWV: body weight variation; AW: atrial weight; RVW: right ventricle weight; LVW: left ventricle weight; AW/BW: atrial-body weight ratio; RVW/BW: right ventricle-body weight ratio; LVW/BW: left ventricle-body weight ratio; AW/tibia: atrial weight-tibia length ratio; RVW/tibia: right ventricle weight-tibia length ratio; LVW/Tibia: left ventricle weight-tibia length ratio; * p<0.05 vs. Control; ‡ p<0.05 vs. ET (two-way ANOVA and Student-Newman-Keuls test).

Analysis of cardiac morphology results showed that intermittent fasting *per se* was associated not only with lower atrial weight (0.063±0.002 vs. 0.053±0.002 g; p=0.006), but also with reduced left ventricle weight, in absolute terms (0.475±0.011 vs 0.420±0.011 mg; p<0.001) and compared to the tibia length. In addition, intermittent fasting reduced heart weight (0.677±0.013 vs. 0.597±0.013 g; p<0.001) ( Table 2 ).

Descriptive measurements of myocardial morphometry are shown in Figure 3 . The combination of intermittent fasting and exercise training resulted in a significantly smaller cellular area in the ETI group compared to the ET and IF groups (control: 248±46; IF: 255±21; PE: 260±30; PIF: 225±26 μm^2^). Considering the frequency distribution of cardiomyocytes, most results were classified within the first two classes, delimited to 327.5 µm^2^. However, the ETI group showed a higher proportion of fibers in the 1^st^class of values (up to 190.1 µm^2^) compared to the other groups (p<0.05) ( Figure 3B ).

**Figure 3:** – (A) Cross-sectional area of the cardiomyocytes (CSA); † p<0.05 versus IF; ‡ p<0.05 vs. ET. Two-way ANOVA and Student-Newman-Keuls test. (B) Frequency distribution of cardiomyocytes according to CSA class. Classes: I (52.7┤190.1 µm^2^), II (190.1┤327.6 µm^2^), III (327.6┤465.0 µm^2^), IV (465.0┤602.4 µm^2^) and V (602.4┤739.9 µm^2^); * p<0.05 vs. Control; † p<0.05 vs. IF; ‡ p<0.05 vs. ET. Goodman test for contrasts within and between multinomial populations. Control, sedentary rats receiving the control diet ad libitum; IF: sedentary rats undergoing intermittent fasting; ET: rats exercising and receiving the control diet ad libitum; ETI: rats exercising while intermittent fasting.

A statistically significant interaction between intermittent fasting and exercise training was obtained in the interstitial collagen fraction analysis (p=0.01). The fraction of interstitial collagen was greater in the ET group compared to control (control: 5.32±1.02; IF: 5.25±0.66; ET: 7.31±2.94; ETI: 4.43±0.79%), while ETI exhibited a lower concentration of interstitial collagen than ET ( Figure 4A-B ).

**Figure 4:** – (A) Myocardial cross-sections stained with picrosirius red according to group. Control (C), sedentary rats receiving the control diet ad libitum; IF, sedentary rats undergoing intermittent fasting; ET: rats exercising and receiving the control diet ad libitum; ETI: rats exercising while intermittent fasting. (B) Interstitial collagen fraction (ICF); * p<0.05 vs. Control; ‡ p<0.05 vs. ET. Two-way ANOVA and Student-Newman-Keuls test.

Tables 3 shows the myocardial expression levels of MAPK-ERK and JNK. No differences were found in the expression of MAPK proteins between the groups (p>0.05).

**Table 3:** – Protein levels of MAPK isoforms in myocardial tissue

Protein	Group			
	Control n=6	IF n=6	ET n=6	ETI n=6
p-ERK/ERK	1.00 ± 0.52	1.42 ± 1.59	1.08 ± 0.48	1.23 ± 0.78
p-ERK/GAPDH	1.00 ± 0.47	1.18 ± 1.19	0.87 ± 0.41	0.86 ± 0.32
ERK/GAPDH	1.00 ± 0.10	0.99 ± 0.23	0.91 ± 0.16	0.91 ± 0.22
p-JNK/JNK	1.00 ± 0.39	1.00 ± 0.35	1.09 ± 0.61	1.03 ± 0.40
p-JNK/GAPDH	1.00 ± 0.13	1.11 ± 0.26	1.10 ± 0.35	1.15 ± 0.23
JNK/GAPDH	1.00 ± 0.46	1.04 ± 0.42	0.94 ± 0.31	0.96 ± 0.32

*Values expressed as mean ± standard deviation. ERK: extracellular signal-regulated kinase; JNK: c-Jun N-terminal kinase. Two-Way ANOVA (p>0.05).*

## Discussion

Restrictive dietary interventions are commonly used to reduce susceptibility to chronic diseases, such as obesity, type 2 diabetes, dyslipidemia, hypertension and cardiovascular diseases. In the present study, intermittent fasting was associated with higher food and energy intake on the days the diet was offered, as well as with lower total caloric intake and lower BW measurements. Higher food intake due to intermittent fasting can be explained by changes in satiety.^29 , 30^

The hypothalamus is an important regulator of body homeostasis and promotes several adjustments, including satiety induction. Changes in the lateral hypothalamus result in aphagia (starvation), whereas alterations in the medial hypothalamus are associated with hyperphagia (increased appetite). As observed here, other studies^11 , 29^ have shown that intermittent fasting increases food intake during feeding days. Likewise, Dorighello et al.^31^ demonstrated that intermittent restriction reduced total calorie intake, in agreement with our findings. The period of exercise training did not affect food or calorie intake in the ETI group. Buthani et al.,^32^ in a study on humans, showed that trained volunteers, even with increased hunger, did not exhibit a significant increase in food intake.

According to our initial hypothesis, exercise training on a treadmill attenuates metabolic disorders and cardiac remodeling induced by intermittent fasting. In addition to reducing energy efficiency, intermittent fasting modified glucose tolerance, which may be associated with better insulin sensitivity. The fact that insulin promotes lipogenic effects in the adipose tissue^33^ may explain why the groups undergoing intermittent fasting did not show significant differences in body adiposity compared to the respective controls ( Table 2 ). Insulin release increases in the presence of greater nutrient availability, such as during the postprandial period. Improvement in insulin sensitivity by adopting an intermittent fasting regime was also found in a recent study on mice.^34^

On the other hand, the combination of intermittent fasting and exercise training reduced body adiposity in the ETI group. Exercise training promotes cardiorespiratory, neural and hormonal adaptations and adjustments.^9 , 15 , 16^ Within the endocrine context, hormone secretion is also altered by exercise. In the study by Evans et al.,^35^ aerobic exercise for 12 months improved insulin sensitivity, with a 19.4% decrease in the area under the oral glucose tolerance curve in older adults. Therefore, the interaction between intermittent fasting and exercise training may have potentiated hormonal effects of insulin metabolism, as supported by the findings obtained for the ET and ETI groups.

Despite glycemic effects, intermittent fasting did not alter functional fitness. Lactate measurement is one of the parameters most used to estimate aerobic capacity and has been effective to describe functional fitness.^22^ The lactate threshold can be defined as the exercise intensity at which the blood lactate concentration suddenly increases.^21 , 22^ In this respect, the running protocol improved functional fitness in the ET and ETI groups, which was supported by lower final lactate values (LacE), less variation in lactate levels and higher speed and distance covered during the final test. Therefore, exercise training was associated with improved aerobic fitness, as previously demonstrated.^16^

Regarding cardiovascular features, exercise training promoted myocardial interstitial remodeling. Intriguingly, intermittent fasting attenuated these exercise-induced effects, as demonstrated by lower values of tissue macro- and microscopic morphometry in the ETI group. Multiple factors stimulate myocardial remodeling, such as nutritional disorders, angiotensin, aldosterone, endothelin, inflammatory cytokines and catecholamines.^36^ Morphological and functional changes occur in response to prolonged exercise training in order to improve cardiac performance, including the blood volume pumped and oxygen supply to peripheral muscles recruited during exercise.^21 , 22^ These adaptive alterations induced by exercise training include left ventricular hypertrophy to compensate for hemodynamic demand and interstitial fibrosis.^36 , 37^ Within this context, increased interstitial collagen found in the ETI group may be an indicator of the physiological ventricular remodeling process, although no myocardial hypertrophy was observed. Indeed, some factors can restrict the accuracy of microscopic morphometry, such as tissue sectioning angle and the heterogeneous contractile state of cardiac fibers.^38^ These factors may have contributed to the lack of detection of cardiac hypertrophy induced by exercise training.

Intermittent fasting resulted in lower values of macro and microscopic morphology, which were not associated with changes of MAPK protein expression. The phenotypic changes induced by these peptides involve protein synthesis and cell growth, triggering hypertrophy and interstitial fibrosis, which may be associated with myocardial remodeling.^13 , 14^ In addition, MAPK activation is also subordinated to the action of multiple growth factors, such as growth hormone and insulin,^24 , 33^ whose secretion is regulated by nutritional behavior. However, it was not possible to verify association between intermittent fasting and changes in MAPK protein expression. In a previous study,^39^ intermittent fasting attenuated cardiac hypertrophy and ventricular dilation in infarcted rats, although it did not alter the gene expression of fetal peptides.

The present findings provide evidence of cardiac remodeling induced by exercise training, which was attenuated by intermittent fasting. However, it is not possible to confirm whether the potential effect of dietary restriction is able to reverse pathological processes, such as arterial hypertension and acute myocardial infarction. Likewise, the impact of other experimental models, such as 25 and 50% calorie restriction,^5 , 7 - 9^ should be further investigated in future studies because they add limitations to the clinical outcomes of the present investigation.

## Conclusion

The combination of intermittent fasting and exercise training intervention is associated with improved glucose tolerance. Exercise training alone promotes myocardial interstitial remodeling, which is attenuated by intermittent fasting.
